# Effect of blastocyst shrinkage on assisted reproductive outcomes: a retrospective cohort study describing a new morphological evaluation of blastocyst pre-vitrification and post-warming

**DOI:** 10.1186/s13048-023-01276-1

**Published:** 2023-09-14

**Authors:** Ayumu Ito, Yukiko Katagiri, Satoko Oigawa, Kenji Amano, Koichiro Ichizawa, Yukiko Tokuda, Mami Unagami, Masato Yoneyama, Takahiro Tsuchiya, Mami Sekiguchi, Mayuko Furui, Kentaro Nakaoka, Nahomi Umemura, Yuko Hayashi, Yuko Tamaki, Koichi Nagao, Masahiko Nakata

**Affiliations:** 1 Department of Obstetrics and Gynecology, Faculty of Medicine, Toho University, 5-21-16, Omorinishi, Ota-Ku, Tokyo, 143-0015 Japan; 2 Department of Obstetrics and Gynecology, Toho University Omori Medical Center, 6-11-1, Omorinishi, Ota-Ku, Tokyo, 143-8541 Japan; 3 Reproduction Center, Toho University Omori Medical Center, 6-11-1, Omorinishi, Ota-Ku, Tokyo, 143-8541 Japan; 4 Department of Urology, Toho University Omori Medical Center, 6-11-1, Omorinishi, Ota-Ku, Tokyo, 143-8541 Japan

**Keywords:** Embryo vitrification, Warming, Blastocyst, Collapse, Expansion, Shrink, Shrinkage, Frozen-thawed blastocyst transfer, Morphological evaluation, Embryo transfer

## Abstract

**Background:**

The failure of frozen-thawed blastocysts to re-expand adequately within a few hours after warming has been reported to have a negative impact on assisted reproductive technology (ART) outcomes. However, the extent to which this failure truly affects ART outcomes has not yet been presented in a manner that is easily understandable to medical practitioners and patients. This study aimed to assess the effects of blastocyst shrinkage on ART outcomes and determine a more effective morphological evaluation approach for use in clinical settings.

**Methods:**

This retrospective observational cohort study of frozen-thawed blastocyst transfer cycles was conducted from April 2017 to March 2022. Overall, 1,331 cycles were eligible for inclusion, of which 999 were good-quality blastocysts (GQB) and 332 were non-good-quality blastocysts (non-GQB). All frozen-thawed blastocyst transfer cycles performed during the specified study period were included in the study. Exclusion criteria were established to mitigate potential sources of bias as these cycles could impact implantations. We calculated rates and age-adjusted odds ratios of implantation, clinical pregnancy, ongoing pregnancy, and live birth of the re-expansion group, which showed sufficient expansion, and shrinkage group, which showed insufficient expansion. We also calculated the implantation, clinical pregnancy, ongoing pregnancy, and live birth rates of the re-expansion and shrinkage groups for each morphological scoring system parameter.

**Results:**

A reduced ART outcome was observed with use of blastocysts with shrinkage after vitrification/warming. The age-adjusted odds ratios for implantation, clinical pregnancy, ongoing pregnancy, and live birth were lower in the shrinkage group than in the re-expansion group.

**Conclusions:**

This study examined the adverse effect of blastocyst shrinkage after warming and recovery culturing on reproductive outcomes in a clinically useful manner by retrospectively examining a substantial number of frozen-thawed embryo transfer cycles. The study findings can possibly reduce concerns regarding over- or under-estimation of blastocyst implantation by allowing providers and patients to refer to the data.

**Supplementary Information:**

The online version contains supplementary material available at 10.1186/s13048-023-01276-1.

## Background

In recent years, advances in cryopreservation and embryo thawing technology have dramatically improved assisted reproductive technology (ART) outcomes. The two primary methods of cryopreservation are slow freezing and vitrification [1]. Slow freezing is a classic method of embryo cryopreservation first proposed by Whittingham et al. [2] and uses a gradually decreasing temperature approach. Vitrification is a rapid cooling method first proposed by Rall and Fahy [3] and reportedly has better ART outcomes than slow-freezing embryos; therefore, it is the mainstream method currently used in embryo cryopreservation [4–8].

Before cryopreservation, blastocysts are evaluated according to their morphological appearance, which is based on the following three parameters: blastocoele expansion, inner cell mass (ICM), and trophectoderm (TE) [9, 10]. Blastocysts shrink during dehydration owing to the addition of cryoprotectants, which are added during cooling in the vitrification process, and then re-expand during rehydration following the removal of cryoprotectants during warming. These shrinkage and re-expansion processes can lead to cell damage, which can subsequently affect blastocyst survival [11]. Furthermore, blastocyst integrity cannot be evaluated accurately, immediately after warming as it is still in a shrunken state. Therefore, it is recommended that blastocysts are cultured for 2–4 h post-warming and evaluated during this time [11].

The ability to re-expand within a few hours of warming has been reported as a strong indicator of blastocyst potential [12], and the implantation and pregnancy rates of insufficiently re-expanded blastocysts are reportedly poor [11, 13, 14]. This indicates that the degree and speed of blastocyst re-expansion post-warming are important factors when evaluating blastocyst integrity and a morphological evaluation before cryopreservation. However, the extent to which the ability to re-expand within a few hours of warming truly impacts ART outcomes has not yet been presented in a manner that is easily understandable to medical practitioners and patients.

The objectives of this study were to clarify the effect of insufficiently re-expanded blastocysts following vitrification/warming on blastocyst implantation, clinical pregnancy, ongoing pregnancy, and live birth rates and to further establish a morphological evaluation process, which considers both pre-vitrification and post-warming findings, for clinical use.

## Results

There were 1,567 frozen-thawed blastocyst transfers during the study period. After screening cases according to the exclusion criteria, 1,331 cycles were eligible for inclusion in the study. Of those cycles, 999 were good-quality blastocysts (GQBs) and 332 were non-GQBs. Blastocyst shrinkage accounted for 15.4% of the cycles (205/1,331). The mean maternal age at oocyte retrieval was 37.2 ± 4.3 years, and the mean body mass index was 22.5 ± 3.8 kg/m^2^. The number of frozen-thawed embryo transfer (ET) cycles consisted of 89 natural and 1,331 hormone replacement cycles. Assisted hatching was performed in 89.0% of the cases (1,183/1,331), and hyaluronic acid-enriched transfer medium (EmbryoGlue®, Vitrolife, Gothenburg, Sweden) was used in 2.7% of the cases (36/1,331). The percentage of blastocyst shrinkage with a re-expansion rate of < 90% at 9–11 min post-warming was 19.9%. The percentage of equilibrated blastocysts with shrinkage that shrunk immediately before ET (shrinkage group) was 15.4%. Age and body mass index were significantly higher in the shrinkage group than in the re-expansion group (*p* < 0.001 and *p* < 0.05, respectively). The probability of GQBs was significantly lower in the shrinkage group than in the re-expansion group (51.2% vs. 79.4%, *p* < 0.001). The background data of patients are presented in Table 1.

**Table 1:** Baseline characteristics of the study participants

Characteristic	All (1,331 cycles)	Re-expasion group (1,126 cycles)	Shrinkage group (205 cycles)	*P* value
Age	38.0 (22–48)	37.0 (22–48)	38.4 (25–48)	< 0.001
BMI (kg/m2)	22.0 (0–38.6)	21.8 (0–37.4)	22.3 (16.9–38.6)	< 0.05
AMH (ng/mL)	2.48 (0.02–46.1)	3.2 (0–18.5)	3.00 (0.05–17.3)	< 0.01
Basal FSH (mIU/mL)	7.0 (0–31.2)	6.9 (0–31.2)	7.2 (0.4–25.6)	N.S
Basal LH (mIU/mL)	4.6 (0–14.8)	4.6 (0–14.8)	4.9 (0–14.5)	N.S
Basal E2 (pg/mL)	33.5 (0–98.4)	33.4 (0–98.4)	34.4 (0–92.2)	N.S
Endometrial thickness (mm)	9.9 (6.2–20.2)	9.9 (6.2–20.2)	9.8 (7.2–17.2)	N.S
Serum E2 value on ET day (pg/mL)	227 (10–1,745)	224 (10–1,745)	234 (26.7–1,585)	N.S
Serum P4 value on ET day (ng/mL)	12.6 (0.11–81.2)	12.7 (1.5–81.2)	12.8 (1.24–34.7)	N.S
Number of natural cycles/hormone replacement therapy cycles (n)	89/1,242	70/1,056	19/186	N.S
GQB (%[n])	75.1 (999)	79.4 (894)	51.2 (105)	< 0.001
Blastocyst shrinkage at 2–11 min after warming (%[n])	19.9 (265)	7.6 (86)	87.3 (179)	< 0.001
Assisted hatching (%[n])	89.0 (1,183)	88.3 (993)	93.2 (191)	< 0.05
Hyaluronic acid-enriched transfer medium	2.7 (36)	2.7 (30)	2.9 (6)	N.S
Infertility causes (total number of causes)	1,484	1,260	438	
Male factor	514 (34.6%)	430 (34.1%)	84 (37.5%)	N.S
Tubal factor	101 (6.8%)	89 (7.1%)	12 (5.4%)	N.S
Cervical factor	16 (1.1%)	15 (1.2%)	1 (0.45%)	N.S
Endometriosis	72 (4.9%)	59 (4.7%)	13 (5.8%)	N.S
Polycystic ovarian syndrome	58 (3.9%)	52 (4.1%)	6 (2.7%)	N.S
Thyroid dysfunction	38 (2.6%)	34 (2.7%)	4 (1.8%)	N.S
Hyperprolactinemia	4 (0.27%)	2 (0.16%)	2 (0.89%)	N.S
Anti-sperm-antibodies	12 (0.81%)	12 (0.95%)	0 (0%)	N.S
Uterine fibroid	48 (3.2%)	42 (3.3%)	6 (2.7%)	N.S
Adenomyosis	5 (0.34%)	5 (0.40%)	0 (0%)	N.S
Uterine malformation	30 (2.0%)	25 (2.0%)	5 (2.2%)	N.S
Diminished ovarian reserve	133 (9.0%)	109 (8.7%)	24 (10.7%)	N.S
Oncofetility	14 (0.94%)	14 (1.1%)	0 (0%)	N.S
Unexplained infertility	438 (29.5%)	371 (29.4%)	67 (29.9%)	N.S

*AMH* anti-müllerian hormone, *BMI* body mass index, *E2* estradiol, *FSH* follicle-stimulating hormone, *GQB* good-quality blastocyst, *LH* luteinising hormone, *P4* progesterone, *N.S.* not significant

### Implantation, clinical pregnancy, ongoing pregnancy, and live birth rates of blastocysts with shrinkage

**Fig. 1:**
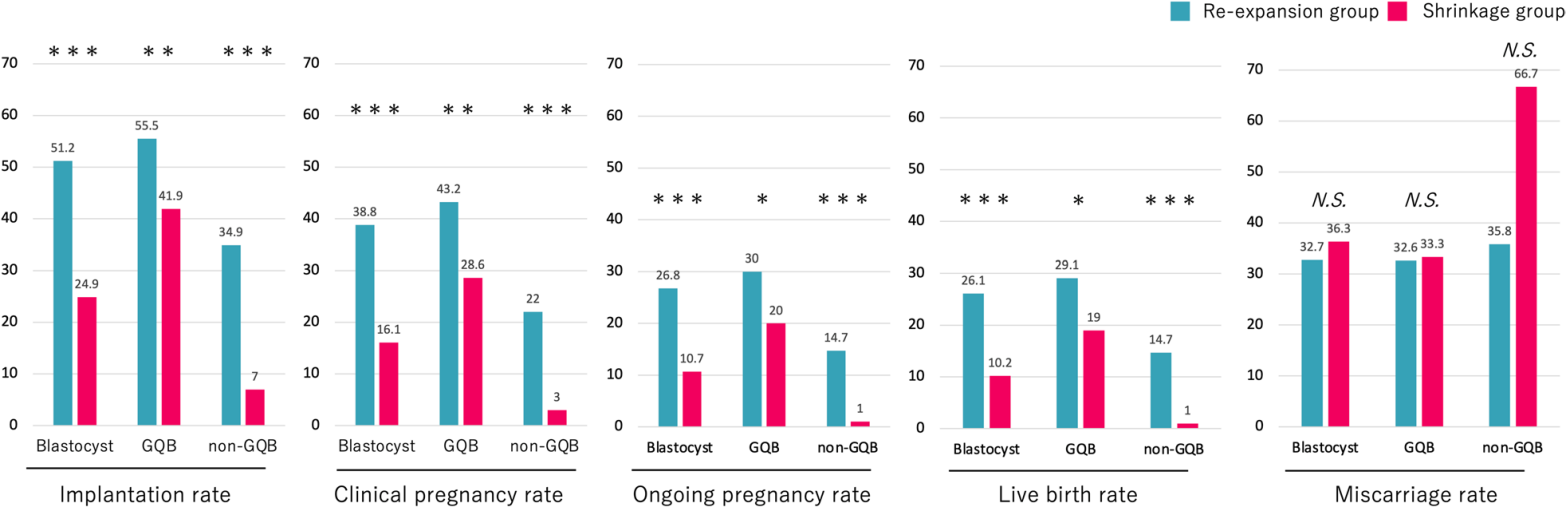
Comparison of assisted reproductive technology outcomes between the re-expansion and shrinkage groups. GQB, good-quality blastocyst; non-GQB, non-good-quality blastocyst; * *p* < 0.05, ** *p* < 0.01, *** *p* < 0.001

The implantation, clinical pregnancy, ongoing pregnancy, and live birth rates of the shrinkage group were all significantly lower than those of the re-expansion group. This trend was similar even when restricted to GQBs or non-GQBs, and all rates tended to be very low (< 10%) in the shrinkage state of non-GQBs (Fig. 1).

**Fig. 2:**
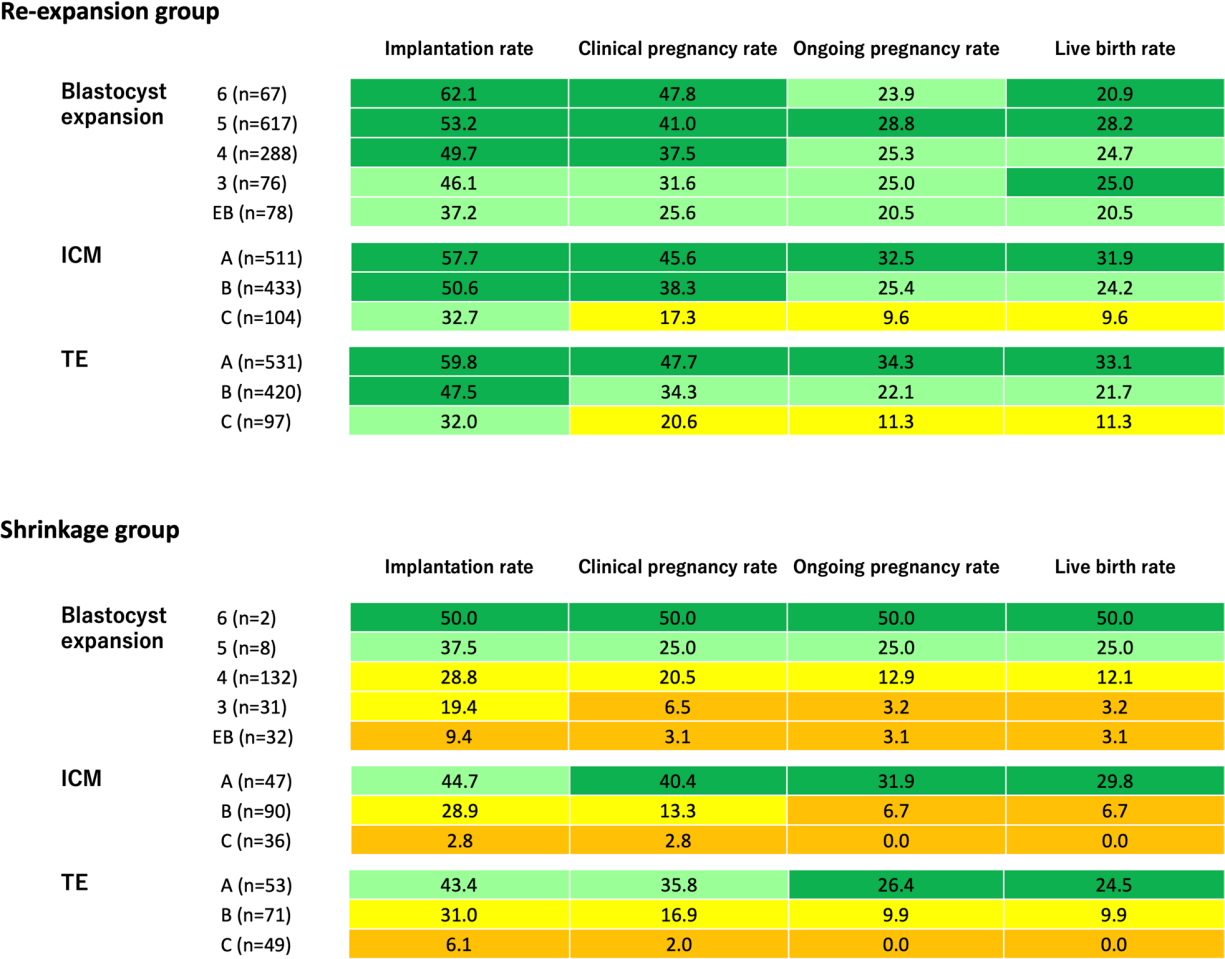
Assisted reproductive technology outcomes of re-expansion and shrinkage blastocysts for each morphological scoring system parameter. For improved visualisation, this figure was colour-coded green, yellow-green, yellow, and orange, with the highest probability number divided into quartiles in ascending order, for blastocoele expansion, trophectoderm (TE), and inner cell mass (ICM), respectively. All units are shown as percentages (%), and the numbers in parentheses represent the number of cycles in the re-expansion or shrinkage groups. EB; early blastocyst

The implantation, clinical pregnancy, ongoing pregnancy, and live birth rates of the re-expansion and shrinkage groups when analysed according to blastocoele expansion, TE, and ICM are shown in Fig. 2. To increase visualisation, these numbers were divided into quartiles, starting with the highest probability value for each, and colour coded. Visualisation was performed for each blastocoele expansion, TE, and ICM. The observed probabilities tended to decrease in the order of the principal morphological scoring system. However, there were some exceptions in cases that had a small number of cycles. In principle, the shrinkage group had a lower probability of all morphological parameters than the re-expansion group. The implantation, clinical pregnancy, ongoing pregnancy, and live birth rates were extremely low (0–9.4%) in the blastocoele expansion early blastocysts (EBs), C in the ICM, and C in the TE of the shrinkage group. However, the implantation rates were noticeably higher (32.0–37.2%) in the re-expansion group.

### Odds ratios for implantation, clinical pregnancy, ongoing pregnancy, and live birth of blastocysts with shrinkage

**Fig. 3:**
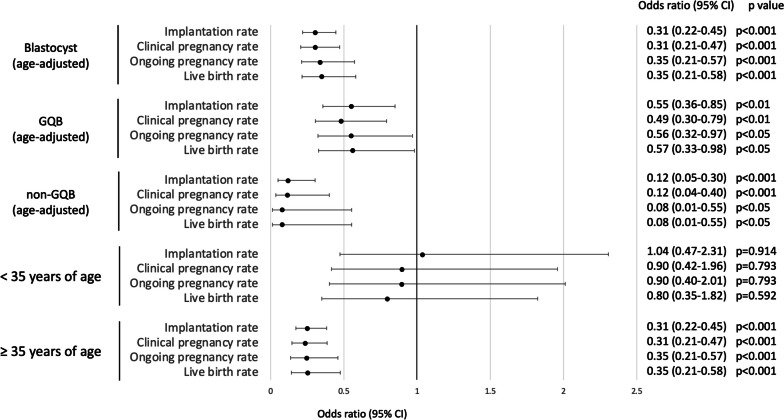
Odds ratios for implantation, clinical pregnancy, ongoing pregnancy, and live birth of blastocysts with shrinkage. GQB; good-quality blastocyst, non-GQB; non-good-quality blastocyst

The age-adjusted odds ratios for implantation, clinical pregnancy, ongoing pregnancy, and live birth in the shrinkage group were 0.31–0.35 (Fig. 3). However, for GQBs, a slightly higher age-adjusted odds ratio of 0.49–0.57 was reported. Conversely, for non-GQBs, the age-adjusted odds ratios were extremely low at 0.08–0.12. The odds ratios by age for the shrinkage group were 0.80–1.0 for those aged < 35 years and 0.31–0.35 for those aged ≥ 35 years. The odds ratios (95% confidence intervals (CI)) for miscarriage in the shrinkage group were 1.33 (0.61–2.91) for all blastocysts (age-adjusted), 1.23 (0.53–2.86) for GQBs (age-adjusted), 3.80 (0.29–50.0) for non-GQBs (age-adjusted), 1.41 (0.41–4.84) for blastocysts from women aged < 35 years, and 1.12 (0.44–2.83) for blastocysts from women aged ≥ 35 years (Additional file 1).

Multivariate logistic regression analysis showed that age (OR, 0.901; 95% CI, 0.871–0.931), non-GQB (OR, 0.388; 95% CI, 0.268–0.561), blastocyst stage reached after day 6 (OR, 0.363; 95% CI, 0.187–0.704), and blastocyst shrinkage post-warming and recovery culturing (OR, 0.389; 95% CI, 0.245–0.618) were independent predictors of clinical pregnancy (Table 2).

**Table 2:** Predictors of clinical pregnancy

Predictor	Odds ratio	95% CI	*P* value	Significance
Age	0.901	0.871–0.931	< 0.001	***
BMI	0.992	0.957–1.029	0.669	
AMH	1.006	0.958–1.056	0.825	
Basal FSH	0.975	0.928–1.025	0.328	
Basal LH	1.048	0.982–1.118	0.161	
Basal E2	1.000	0.993–1.006	0.882	
Endometrial thickness	1.037	0.968–1.110	0.300	
Serum E2 value on ET day	1.000	0.999–1.001	0.699	
Serum P4 value on ET day	1.022	1.001–1.043	< 0.05	*
Non-GQB	0.388	0.268–0.561	< 0.001	***
Blastocyst stage reached after day 6	0.363	0.187–0.704	< 0.01	**
Blastocyst shrinkage post-warming and recovery culturing	0.389	0.245–0.618	< 0.001	***
Assisted hatching	0.762	0.510–1.139	0.185	
Hyaluronic acid-enriched transfer medium	1.097	0.469–2.564	0.831	

*AMH* anti-müllerian hormone, *BMI* body mass index, *E2* estradiol, *FSH* follicle-stimulating hormone, *GQB* good-quality blastocyst, *LH* luteinising hormone, *P4* progesterone, *95% CI* 95% confidence interval, * *p* <0.05, ** *p*<0.01, *** *p*<0.001

### Probability of blastocyst shrinking after warming and recovery culturing

The probability of blastocyst shrinking after warming and recovery culturing was significantly higher in the non-GQB group than in the GQB group (30.1% vs. 10.5%), in women aged ≥ 35 years than in those aged < 35 years (17.8% vs. 8.4%) and in embryos that became blastocysts after day 6 than in those at day 5 or earlier (27.0% vs. 14.5%) (Additional file 2).

## Discussion

The results of this study indicate that blastocyst shrinkage significantly reduces ART outcomes such as implantation, clinical pregnancy, ongoing pregnancy, and live birth rates. The likelihood of experiencing blastocyst shrinkage was particularly high in non-GQBs, in blastocysts from women aged ≥ 35 years and in blastocysts that developed after day 6.

Implantation, pregnancy, and live birth rates were significantly associated with the presence or absence of blastocyst shrinkage after warming and recovery culturing. Blastocyst re-expansion post-warming is reportedly associated with increased live birth rates and improved implantation and pregnancy rates [11, 15]. Shu et al. additionally stated that a minimum number of surviving cells are required for blastocyst re-expansion [11]. Furthermore, blastocyst expansion is thought to be caused by the influx of extracellular fluid into the blastocyst cavity through the aquaporin channel, which increases the sodium ion (Na +) concentration in the blastocyst cavity through the interaction of sodium and potassium ions (K +) with ATPase (Na + /K + -ATPase) present in the TE [16–18]. Human and bovine studies have shown that the glucose consumption of blastocysts with re-expansion is higher than that of non-expanded blastocysts after cryopreservation and thawing [19, 20], which is consistent with the involvement of Na + /K + -ATPase in blastocyst expansion. Therefore, sufficient blastocyst re-expansion after freezing and thawing is a good morphological marker in selecting good embryos [19, 20].

The likelihood of experiencing blastocyst shrinkage was significantly high in non-GQBs, in blastocysts from women aged > 35 years, and in blastocysts that reached full blastulation after 6 days. In fact, it has been reported that the odds ratio for the insufficient blastocyst re-expansion after warming was significantly higher in embryos with poor morphological quality (odds ratio [95% CI]: 19.54 [8.39–45.50]) and in embryos taking 7 days to reach a full blastocyst (odds ratio [95% CI]: 3.19 [1.23–8.29] [14]. One potential explanation for this is that these blastocysts contain many aneuploid embryos. Huang et al. reported that the average expansion rate of euploid blastocysts was significantly higher than that of aneuploid blastocysts and that aneuploidy resulting in universal cellular deficits could impair blastocoele expansion [21]. The relationship between maternal age or blastocyst formation date and the euploid rate has been extensively studied. This relationship suggests that the euploid rate of blastocysts decreases with higher maternal age and also with slower full blastocyst formation time from day 5 to 7 [22]. This supports the hypothesis that blastocysts with shrinkage are likely to become aneuploid embryos. Furthermore, an association between embryo ploidy and embryo morphological quality has been noted. Wang et al. reported a significant association between embryonic ploidy and blastocoele expansion, ICM, and TE [23]. Therefore, we considered that a GQB, which is a morphologically good-quality embryo, contains more euploid embryos than a non-GQB. The age-adjusted odds ratio of the blastocyst shrinkage for implantation, clinical pregnancy, ongoing pregnancy, and live birth rates suggested that the impact of blastocyst shrinkage is stronger for non-GQBs than for GQBs in ART outcomes. However, Cimadomo et al. [14] have reported that the likelihood of achieving a live birth is significantly lower for blastocysts that are of poor quality and slow to develop, even if they are euploid embryos. This suggests that the morphological evaluation of blastocysts, both before vitrification and after warming, may have an impact on the outcome of ART, regardless of their euploidy.

As shown in Fig. 2, the impact of blastocyst shrinkage also tended to increase with the decreased quality of all morphological scoring system parameters (blastocoele expansion, ICM, and TE). This re-expansion heterogeneity was thought to be due to the inability to discern whether the shrinkage state immediately before ET was a universal change due to cellular deficits caused by aneuploidy or variable physiological changes. We speculated that the shrinkage state of GQBs was most probably due to variable physiological changes and that for non-GQBs, the shrinkage state was most probably due to universal changes due to cellular damage. The absence of significant differences in the age-adjusted odds ratios for abortion rates due to blastocyst shrinkage suggests that the shrinkage state of non-GQBs capable of implantation may be due to variable physiological changes. However, non-GQBs with shrinkage will often not implant. In summary, for GQBs of women aged < 35 years and those that have reached the blastocyst stage by day 5, there was no significant concern about a prominent decline in ART outcomes with respect to shrinkage immediately before ET.

A significant strength of this study is that it examines the adverse effect of blastocyst shrinkage post-warming and recovery culturing on reproductive outcomes by retrospectively examining a substantial number of frozen-thawed ET cycles. These findings can potentially reduce the concern of over- or under-estimation of blastocyst implantation by allowing both providers and patients to refer to the data. In addition, this study provides evidence for identifying embryos that may shrink after warming and recovery culture, which may help in the selection criteria for blastocyst freezing. However, it is important to note that this study was conducted at a single centre utilising a consistent protocol and that the generalisability of the study results may be limited. Another limitation of this study is that time-lapse imaging was not used, and the embryos could not be observed between 9–11 min post-warming and immediately before ET. It is thus not possible to distinguish whether blastocysts with shrinkage observed immediately before ET was an embryo that insufficiently re-expanded at 9–11 min post-warming and remained shrunken or whether it re-expanded but shrunk again. Time-lapse imaging may provide further insights into the physiological and clinical significance of the shrinkage state. Furthermore, the timing of observation for blastocyst re-expansion assessment involved a rather wide range (2–7 h post-warming, depending on the degree of blastocoele expansion), which may introduce bias. Unfortunately, we were unable to eliminate this bias owing to the retrospective nature of the study. Another limitation of this study is the difficulty in abandoning ET, even if the embryo is found to be in a shrunken state immediately before ET. Therefore, double ET may be considered for cases where the ET is likely to result in blastocyst shrinkage, such as with a non-GQB, blastocysts from women aged > 35 years, and blastocysts that have reached full blastocyst after day 6. Furthermore, by identifying early morphological markers of the embryo that can affect implantation and pregnancy outcomes, such as the shrinkage rate at cryopreservation and re-expansion rate after warming, enhanced embryo selection will be possible without abandoning the transfer cycle. Prospective studies using time-lapse imaging are therefore critical for this purpose. It should also be noted that even though the implantation and pregnancy rates of blastocysts with shrinkage are lower than those of blastocysts with re-expansion, these odds are never zero, and pregnancy is possible.

## Conclusions

It is known that blastocyst shrinkage post-warming and recovery culturing reduces ART outcomes. In addition to the classic morphological evaluation before cryopreservation, a more detailed evaluation can be performed by combining morphological evaluation post-warming, which will allow enhanced embryo selection and result in improved patient outcomes.

## Methods

### Aim of the study

This study aimed to assess the effects of blastocyst shrinkage on ART outcomes and determine a more effective morphological evaluation approach for use in clinical settings.

### Study design and patient population

This retrospective observational cohort study of frozen-thawed blastocyst transfer cycles was conducted from April 2017 to March 2022 at the Reproduction Center of Toho University Omori Medical Center, Tokyo, Japan.

All frozen-thawed blastocyst transfer cycles performed during the study period were eligible for inclusion. However, exclusion criteria were established to mitigate potential bias sources that could impact implantations. Therefore, cycles involving multiple ETs, transfers based on endometrial receptivity analysis (ERA®, Igenomix, Valencia, Spain) results, transfers combined with platelet-rich plasma therapy, and transfers of embryos that underwent pre-implantation genetic testing for aneuploidy were excluded from the study.

### Embryo culture, morphological evaluation of blastocysts, and embryo vitrification protocol

Oocytes retrieved during the controlled ovarian stimulation cycle were cultured in a fertilisation medium (Insemination Medium, Nakamedical Inc., Tokyo, Japan) at 6% CO_2_ and 5% O_2_ at 37 °C and fertilised using in vitro fertilisation (IVF) or intracytoplasmic sperm injection. Zygotes were cultured individually in 60-μL droplets (ONESTEP Medium, Nakamedical, Tokyo, Japan) after intracytoplasmic sperm injection. During IVF, on the day after oocyte retrieval, zygotes were denuded and fertilisation was confirmed by the presence of two pronuclei, at which point the zygotes were placed in individual droplets of 60 μL (ONESTEP Medium). In intracytoplasmic sperm injection, fertilisation was only confirmed with no further intervention. On day 5 after oocyte retrieval, embryos were evaluated using an inverted microscope and transferred to individual droplets (ONESTEP Medium) for culturing toward the blastocyst stage.

Blastocysts were evaluated according to the morphological scoring system of the blastocyst, which was introduced by Gardner and Schoolcraft [9, 10] and assessed for blastocoele expansion, ICM, and TE appearance. Blastocoele expansion degrees 1 and 2 were defined as EBs.

All blastocysts were cryopreserved on day 5, 6, or 7 unless otherwise requested by the patient. Embryos that had developed at least to the early blastocyst stage on day 5 were frozen. Embryos that did not reach the early blastocyst stage were monitored every day up to a maximum of 7 days and frozen if they reached the early blastocyst stage. At least two of the four experienced embryologists (S.O./K.A./K.I./Y.T.) evaluated the blastocysts using an inverted microscope and recorded the data.

Embryo vitrification was performed using vitrification media (VT507) (Kitazato Corporation, Shizuoka, Japan). Embryo equilibrations were performed in an equilibration solution containing 7.5% ethylene glycol, 7.5% dimethyl sulfoxide, hydroxypropyl cellulose (HPC), and gentamicin at room temperature (25–27℃) for 10–14 min. Vitrification was performed using a vitrification solution containing 15% ethylene glycol, 15% dimethyl sulfoxide, 0.5 mol/L sucrose, HPC, and gentamicin within a minute, at which point embryo shrinkage was confirmed. The embryos were then pipetted, and a minimal amount of vitrification solution was placed at the tip of the embryo.

The embryo was placed on a Cryotop sheet (Kitazato Corporation, Shizuoka, Japan), which was immediately placed in liquid nitrogen.

### Embryo thawing protocol and definition of blastocyst shrinkage post-warming

Embryo thawing was performed using a thawing kit (VT508) (Kitazato Corporation). The Cryotop sheet was removed from the liquid nitrogen and quickly placed in a thawing solution containing 1.0 mol/L sucrose and gentamicin at 37 °C for warming. After 1 min, embryos were placed in a diluent solution containing 0.5 mol/L sucrose, HPC, and gentamicin for 3 min at room temperature (25–27℃). Embryos were washed with a small amount of the diluent solution mixed with a washing solution containing HPC and gentamicin for 5 min and then washed with a washing solution containing HPC and gentamicin for 1 min at room temperature. Embryos were then cultured for recovery at 37 °C for 2–7 h depending on the degree of blastocoele expansion before ET. The variable range in recovery time was owing to the need for longer incubation periods for embryos with smaller degrees of blastocoele expansion.

Blastocyst shrinkage was assessed 9–11 min post-warming and 2–7 h after recovery culturing, which was immediately before ET. Blastocyst shrinkage was defined as a blastocyst that had a re-expansion rate (embryo area/area inside the zona pellucida) < 90%; blastocyst re-expansion was defined as one whose re-expansion rate was > 90% (Additional file 3). Blastocyst shrinkage was assessed and recorded by at least two of the four experienced embryologists (S.O./K.A./K.I./Y.T.) using an inverted microscope.

### Frozen-thawed blastocyst transfer cycle protocol and outcome definitions

In the natural cycle, follicle growth was monitored, ovulation was predicted using ultrasonography and blood tests, and ET was performed 5 days after the predicted ovulation date. One of four natural transvaginal progesterone suppositories (Lutinus Vaginal Tablets, Ferring Pharmaceuticals, Tokyo, Japan; Luteum Vaginal Suppositories, Aska Pharmaceutical Co., Ltd., Tokyo, Japan; OneCrinone vaginal gel, Merck Biopharma, Tokyo, Japan; UTROGESTAN vaginal capsules 200 mg, Fuji Pharma, Tokyo, Japan) was started 1 day after the predicted ovulation date.

In the hormone replacement cycle, a transdermal oestrogen patch (Estrana tape, Hisamitsu Pharmaceutical Co., Inc., Saga, Japan) was administered from day 3 of menstruation. After confirming that the endometrium was at least 8 mm in principle, transvaginal progesterone treatment was started, and ET was performed 5 days after natural transvaginal progesterone administration.

We suspected implantation when serum hCG levels were ≥ 5 mIU/mL 10 days after ET. Clinical pregnancy was confirmed by the presence of a gestational sac on transvaginal ultrasound between 4 and 6 weeks of gestation. We confirmed ongoing pregnancy at 12 weeks of gestation. Live births after 22 weeks of gestation were defined as live births, and we were informed via correspondence when patients delivered at other hospitals. Miscarriage was defined as delivery before or at 22 weeks after clinical pregnancy confirmation.

### Effect of blastocyst shrinkage on implantation and pregnancy

We compared implantation, clinical pregnancy, ongoing pregnancy, and live birth rates between the re-expansion group, which expanded sufficiently, and the shrinkage group, which expanded insufficiently, after warming and recovery culturing. We then evaluated the blastocysts from these groups separately to identify GQBs and non-GQBs. GQBs were defined as having a grade of at least 3BB, including 3/4/5AA, AB, BA, or BB, based on the morphological scoring system of the blastocyst introduced by Gardner and Schoolcraft [9, 10]. The remaining blastocysts, including degrees 1 and 2 of blastocoele expansion or C of ICM or TE, were defined as non-GQBs.

We calculated the implantation, clinical pregnancy, ongoing pregnancy, and live birth rates of the blastocysts with re-expansion and shrinkage for each morphological scoring system parameter (blastocoele expansion, TE, and ICM) as indicators of expectations for implantation and pregnancy after ET in clinical practice.

We calculated the adjusted odds ratios for implantation, clinical pregnancy, ongoing pregnancy, and live birth of blastocysts with shrinkage after adjusting for female age to eliminate its effect on implantation and pregnancy. We further calculated the adjusted odds ratios of GQBs and non-GQBs. The unadjusted odds ratios were calculated separately for each age group considering a maternal age of < 35 years and ≥ 35 years. Moreover, a multivariate logistic regression analysis was performed to evaluate predictors that could influence clinical pregnancy rates, including factors such as blastocyst shrinkage after warming and the recovery culturing.

We determined the probability of blastocyst shrinkage with insufficient re-expansion post-warming between the GQB and non-GQB groups, between embryos of women aged < 35 years and ≥ 35 years, and between embryos that reached the blastocyst stage at day 5 and those that reached it after day 6.

### Statistical analyses

All statistical analyses were performed using SPSS statistical analysis software (version 28; IBM Corp., Armonk, NY, USA). Comparisons of means between the groups were performed using the t-test for parametric data and the Mann–Whitney U test for nonparametric data. Comparisons of proportions were performed using the chi-square test and Fisher’s exact probability test. Odds ratios are expressed as 95% confidence intervals (CIs). The Mantel–Haenszel test was used to calculate age-adjusted odds ratios. Statistical significance was defined as *p* < 0.05.

No statistical sample size calculations were conducted. However, post hoc analyses revealed that sample sizes of 1,126 and 205 for the re-expansion and shrinkage groups, respectively, yielded 100% power to detect a difference in the odds ratio for implantation, clinical pregnancy, ongoing pregnancy, and live birth rates at 26.3%, 22.7%, 16.0%, and 16.0%, respectively. This determination was made utilising a two-group chi-square test with a two-sided significance level of *p* < 0.05 for percentage change.

### Ethical approval

This study was approved by the Ethics Committee of Toho University Omori Medical Centre (approval no. M22165). Information regarding the study was published in an opt-out format on the hospital website, allowing potential research participants the opportunity to refuse participation. The need for written informed consent was waived.

### Supplementary Information

**Additional file 1.** Odds ratios for miscarriage of blastocysts with shrinkage. Odds ratios for miscarriage of the shrinkage group compared to re-expansion group. GQB; good-quality blastocyst, non-GQB; non-good-quality blastocyst, CI; confidence interval.

**Additional file 2.** Probability of blastocyst shrinking after warming and recovery. The probability of blastocyst shrinkage after warming and recovery culturing was compared by dividing it according to the morphological quality of blastocysts and maternal age. GQB, good-quality blastocyst; non-GQB, non-good-quality blastocyst; D5, embryos that became blastocysts at D5, > D6; embryos that became blastocysts after D6, *** *p* < 0.001.

**Additional file 3. **Example of the calculation method of the blastocyst re-expansion rate. The re-expansion rate was calculated as the area of the embryo (white line)/area inside the zone of transparency (red line) × 100 and was calculated using the ImageJ software (ImageJ, U. S. National Institutes of Health, Bethesda, Maryland, USA) on a photograph of the blastocyst taken just before transfer (A) 100% re-expansion rate (B) 78.4% re-expansion rate.

## Data Availability

The datasets used and analysed during the current study are available from the corresponding author on reasonable request.

## Abbreviations

ART: Assisted Reproductive Technology
CI: Confidence Interval
EB: Early Blastocysts
ET: Embryo Transfer
GQB: Good-Quality Blastocyst
HPC: Hydroxypropyl Cellulose
ICM: Inner Cell Mass
IVF: In Vitro Fertilisation
TE: Trophectoderm
