# Correction: Effect of blastocyst shrinkage on assisted reproductive outcomes: a retrospective cohort study describing a new morphological evaluation of blastocyst pre-vitrification and post-warming

**DOI:** 10.1186/s13048-024-01439-8

**Published:** 2024-05-22

**Authors:** Ayumu Ito, Yukiko Katagiri, Satoko Oigawa, Kenji Amano, Koichiro Ichizawa, Yukiko Tokuda, Mami Unagami, Masato Yoneyama, Takahiro Tsuchiya, Mami Sekiguchi, Mayuko Furui, Kentaro Nakaoka, Nahomi Umemura, Yuko Hayashi, Yuko Tamaki, Koichi Nagao, Masahiko Nakata

**Affiliations:** 1 Department of Obstetrics and Gynecology, Faculty of Medicine, Toho University, 5-21-16, Omorinishi, Ota-Ku, Tokyo, 143-0015 Japan; 2 Department of Obstetrics and Gynecology, Toho University Omori Medical Center, 6-11-1, Omorinishi, Ota-Ku, Tokyo, 143-8541 Japan; 3 Reproduction Center, Toho University Omori Medical Center, 6-11-1, Omorinishi, Ota-Ku, Tokyo, 143-8541 Japan; 4 Department of Urology, Toho University Omori Medical Center, 6-11-1, Omorinishi, Ota-Ku, Tokyo, 143-8541 Japan

**Correction:** ***J Ovarian Res*** **16, 192 (2023)**

10.1186/s13048-023-01276-1

Following publication of the original article [1], the authors corrected the first author’s name and Table 2. See below table for the correct data.

**Table:** 

Incorrect	Correct
The first author’s name was Ayumo Ito	The correct author name is Ayumu Ito
In table 2 the odds ratio for BMI is currently listed as 0.669	The accurate value should be 0.992

The original article has been corrected.
