# Supplementary figures and images for: Effect of blastocyst shrinkage on assisted reproductive outcomes: a retrospective cohort study describing a new morphological evaluation of blastocyst pre-vitrification and post-warming
Source: J Ovarian Res. 2023 Sep 14;16:192. doi: 10.1186/s13048-023-01276-1 (PMC10503151; doi:10.1186/s13048-023-01276-1)

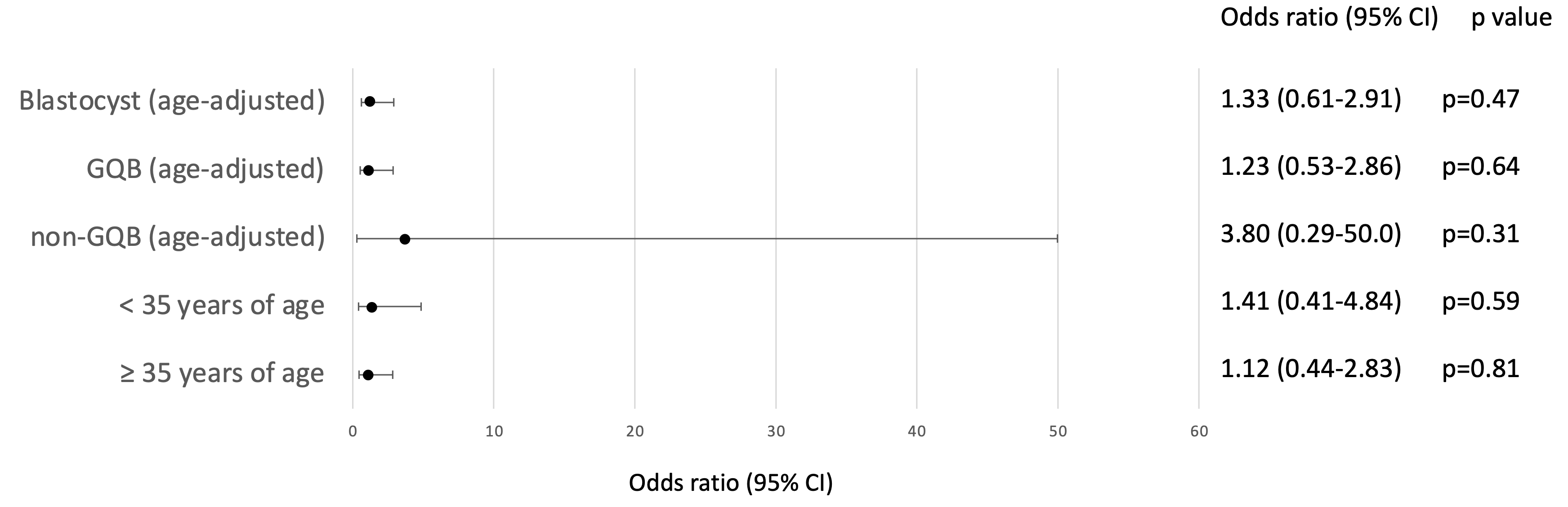

Supplement: Supplementary file 1 — Additional file 1. Odds ratios for miscarriage of blastocysts with shrinkage. Odds ratios for miscarriage of the shrinkage group compared to re-expansion group. GQB; good-quality blastocyst, non-GQB; non-good-quality blastocyst, CI; confidence interval. [file 13048_2023_1276_MOESM1_ESM.tiff]

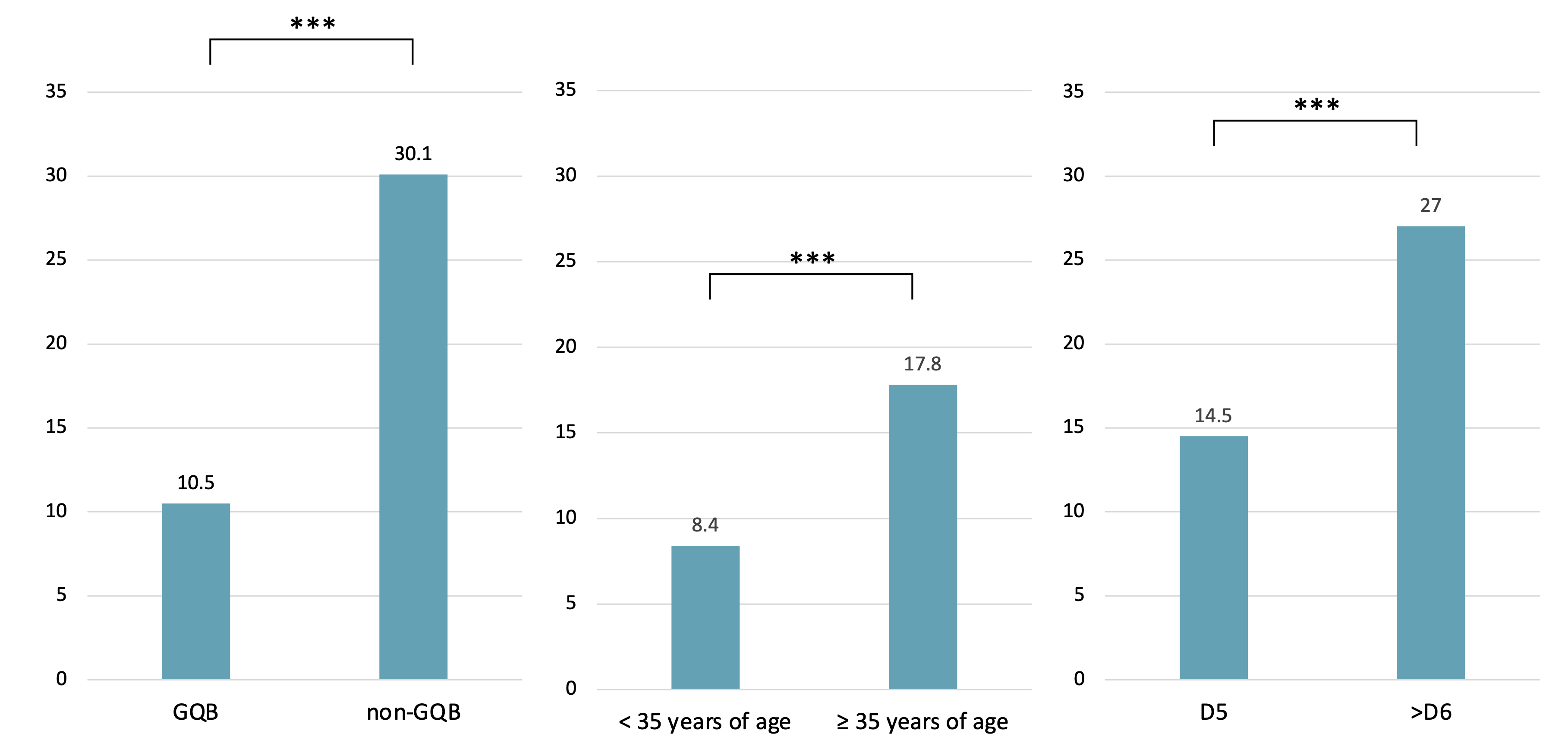

Supplement: Supplementary file 2 — Additional file 2. Probability of blastocyst shrinking after warming and recovery. The probability of blastocyst shrinkage after warming and recovery culturing was compared by dividing it according to the morphological quality of blastocysts and maternal age. GQB, good-quality blastocyst; non-GQB, non-good-quality blastocyst; D5, embryos that became blastocysts at D5, > D6; embryos that became blastocysts after D6, *** p < 0.001. [file 13048_2023_1276_MOESM2_ESM.tiff]

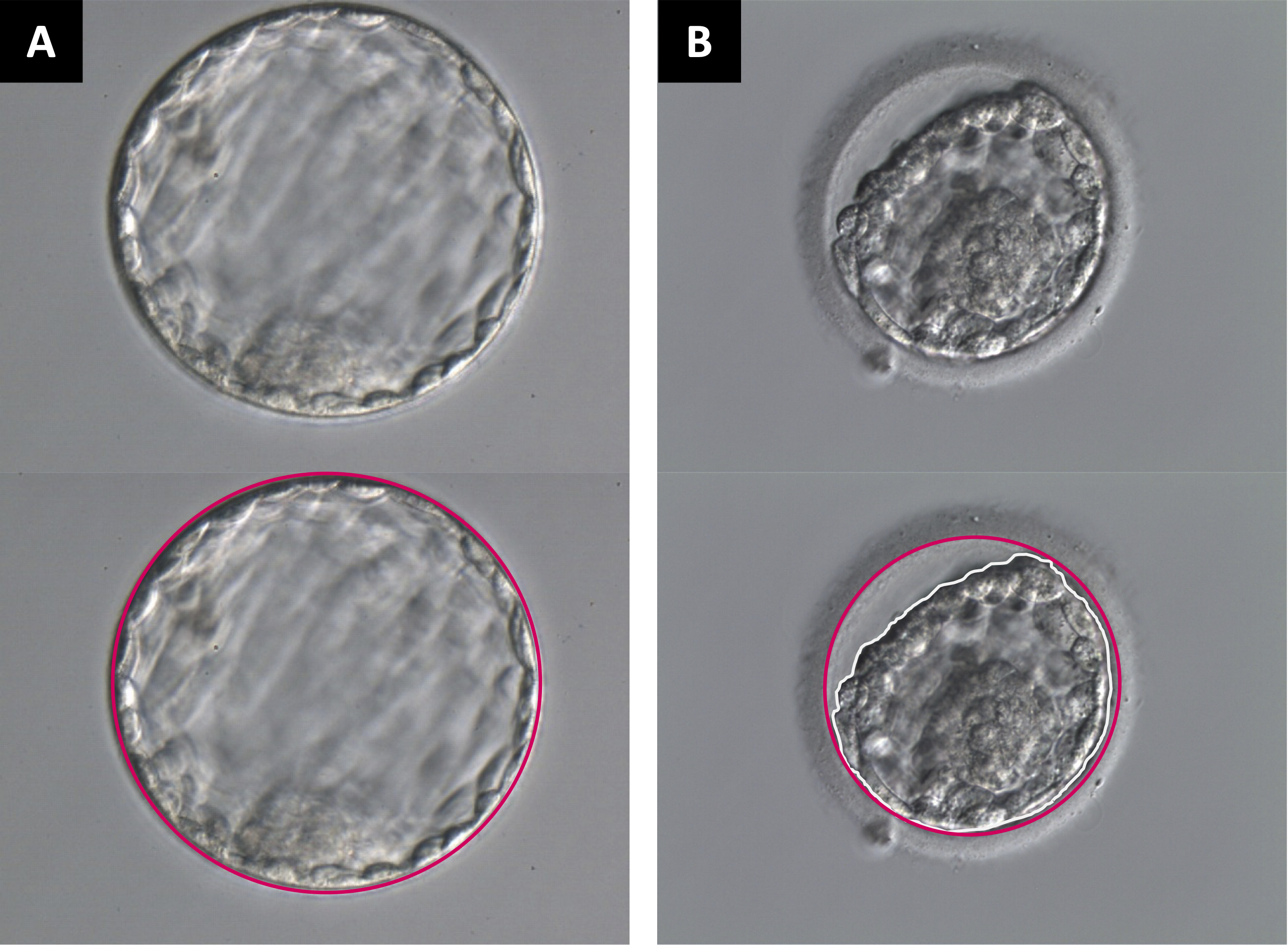

Supplement: Supplementary file 3 — Additional file 3. Example of the calculation method of the blastocyst re-expansion rate. The re-expansion rate was calculated as the area of the embryo (white line)/area inside the zone of transparency (red line) × 100 and was calculated using the ImageJ software (ImageJ, U. S. National Institutes of Health, Bethesda, Maryland, USA) on a photograph of the blastocyst taken just before transfer (A) 100% re-expansion rate (B) 78.4% re-expansion rate. [file 13048_2023_1276_MOESM3_ESM.tiff]
